# Effects of Home-Based Computerized Cognitive Training in Community-Dwelling Adults With Mild Cognitive Impairment

**DOI:** 10.1109/JTEHM.2023.3317189

**Published:** 2023-09-19

**Authors:** Ji Soo Baik, Ji Hong Min, Sung-Hwa Ko, Mi Sook Yun, Byunghoon Lee, Nae Yoon Kang, Byeongil Kim, Hyunsuk Lee, Yong-Il Shin

**Affiliations:** Research Institute for Convergence of Biomedical Science and Technology, Pusan National University Yangsan Hospital 194197 Yangsan 50612 Republic of Korea; Department of Rehabilitation MedicinePusan National University Yangsan Hospital 194197 Yangsan 50612 Republic of Korea; Department of Rehabilitation MedicineThe Graduate School of MedicinePusan National University 34996 Yangsan 50612 Republic of Korea; Department of Rehabilitation MedicinePusan National University Hospital 220312 Busan 46241 Republic of Korea; Woorisoft Daegu 42781 Republic of Korea

**Keywords:** Cognitive function, memory, community dwelling, aged, computer-assisted instruction

## Abstract

Objective: There is a growing importance for the home-based (HB) support services, and computerized cognitive training (CCT) has been reported as an effective intervention for cognitive impairment. However, there is still a need for further verification of the effect of HB-CCT. This study aimed to determine the effectiveness of HB-CCT on the cognitive function of community-dwelling adults with mild cognitive impairment (MCI) as well as safety in its use. Methods: Fifty community-dwelling adults with MCI were included, of which 25 each were randomized to either HB-CCT or control groups. Evaluations of comprehensive cognition, memory, attention, language, executive function, and depression were performed before and after the intervention, including three times a week for eight weeks in the intervention group and eight weeks apart with no intervention in the control group. Results: In baseline and post-evaluation comparisons, the HB-CCT group showed significant improvements, while the control group did not show significant changes. Statistically significant variations were noted between the HB-CCT and control groups in all post-intervention evaluations relative to baseline. Additionally, no side effects were observed. Conclusion: Beneficial effects on cognition and depression were noted in the intervention group compared with the control group, suggesting that HB-CCT may be a positive tool for cognitive improvement in adults with MCI.

## I. Introduction

The development of medical technology has increased life expectancy; however, cognitive decline remains an issue, as the number of older adults with Alzheimer’s disease (AD) or dementia continues to increase with aging populations [1], [2]. The expected annual total medical expenditure per person with dementia is expected to increase as the number of patients with dementia increases [3]. In addition, cognitive decline is considered a major public health problem as it increases the levels of depression and lowers the quality of life for affected individuals and those around them [4], [5]. Returning to pre-onset states once dementia occurs is difficult [6], [7]. Therefore, the prevention of dementia or of cognitive decline is essential [6], [8].

Mild cognitive impairment (MCI), an early stage of dementia, is defined as significant declines in cognition compared with individuals of the same age and similar educational levels [9]. Patients with MCI have a higher risk of dementia compared with healthy older adults [10], [11]. In addition, prognoses of MCI are more positive than those of dementia. Therefore, preventing the development of dementia in adults with MCI is critical [12].

Various studies on the prevention of cognitive decline have been conducted [13], [14], [15]. Among them, there is a growing interest in cognitive therapy using computers, tablets, or smartphones, due to the advantages of user convenience, ease of accessibility, and minimal spatial restrictions [16], [17]. These therapies are collectively known as computerized cognitive training (CCT), with the term first being coined in a 1992 study of students with learning disabilities [18]. As the name suggests, CCT is a method of converting cognitive intervention programs into software formats to train cognition through computerized devices. CCT techniques have fewer side effects, are able to accurately and continuously record data, and are able to create interest and improve concentration [12].

Representative CCTs, including CogReHab, COMCOG, Captain’s Log, Rehab.com, and Cog-med, are mainly used in hospitals, which may limit patient accessibility [12], [16], [19]. Remote treatment via at-home or community-based CCTs may increase accessibility and autonomy in the cognitive training of adults with MCI [20]. The importance of home-based support services has been increasingly highlighted, with the number studies on at-home and community center CCTs increasing [21]. However, further verification of the feedback mechanisms in treatments and the effects on cognitive functioning are required [22]. Thus, this study aimed to examine the effectiveness and safety of portable home-based CCT (HB-CCT) using tablet personalized computers (PCs) on the cognitive functioning of community-dwelling adults with MCI.

## II. Methods

### A. Study Design and Participants

This single-blinded, randomized control pilot study was conducted between 22 April 2020–2 August 2021. The study was approved by the Institutional Review Board of Pusan University Yangsan Hospital (IRB no. 02-2019-018) and registered on Clinictrials.gov (NCT05275153). All participants provided written informed consent prior to initial evaluations.

Participants were community-dwelling adults aged $\ge55$ years with MCI [23]. Adults with MCI were defined as individuals with scores $\ge16$ but < 23 on the Korean-Montreal Cognitive Assessment (MoCA) [24] as well as scores $\ge24$ on the Korean-Mini Mental State Examination [25]. Evaluations were conducted by a neutral party, typically an occupational therapist. Those diagnosed by a neurologist with dementia according to the criteria of neurocognitive disorders (regarding the impact of cognitive impairment on independent functioning in daily life) [26], [27] were excluded from the study. Additionally, patients with a history of depression or neurological conditions and those currently diagnosed with such conditions were excluded.

Individuals were recruited through recruitment notices at university hospitals and community centers for senior citizens in Yangsan-si city, Gyeongsangnam-do province. The required sample size was estimated using $\text{G}^{\ast} $Power (version 3.1.9.4) for analysis of covariance (ANCOVA) [28]. To detect a previous research effect size (f = 0.36) for treatment effects for primary outcomes with conventional $\alpha $ and power levels ( $\alpha $ = 0.05 and $\beta $ = 0.80), a minimum total sample size of 48 was required [29]. Therefore, we recruited 55 participants, five of which were excluded during screening for various reasons, including not meeting the inclusion criteria (n = 2), not having internet access via a computer at home (n = 2), and refusing to participate (n = 1). Ultimately, a total of 50 individuals with MCI participated in the study.

Participants were allocated to either the HB-CCT or control groups by a computer-generated random number list, with 25 participants randomly designated to each group. The HB-CCT intervention in the HB-CCT group was performed three times a week over an 8-week period for a total of 24 occurrences [30], [31], [32]. In this study, the compliance rate of participation was 80%, and participants who did not attend at least 20 of the 24 intervention sessions were considered dropouts. Additionally, concomitant prohibited medications or treatments were not restricted, and ongoing medications or treatments remained unchanged from the screening day to the end of the study period.

**FIGURE 1.**
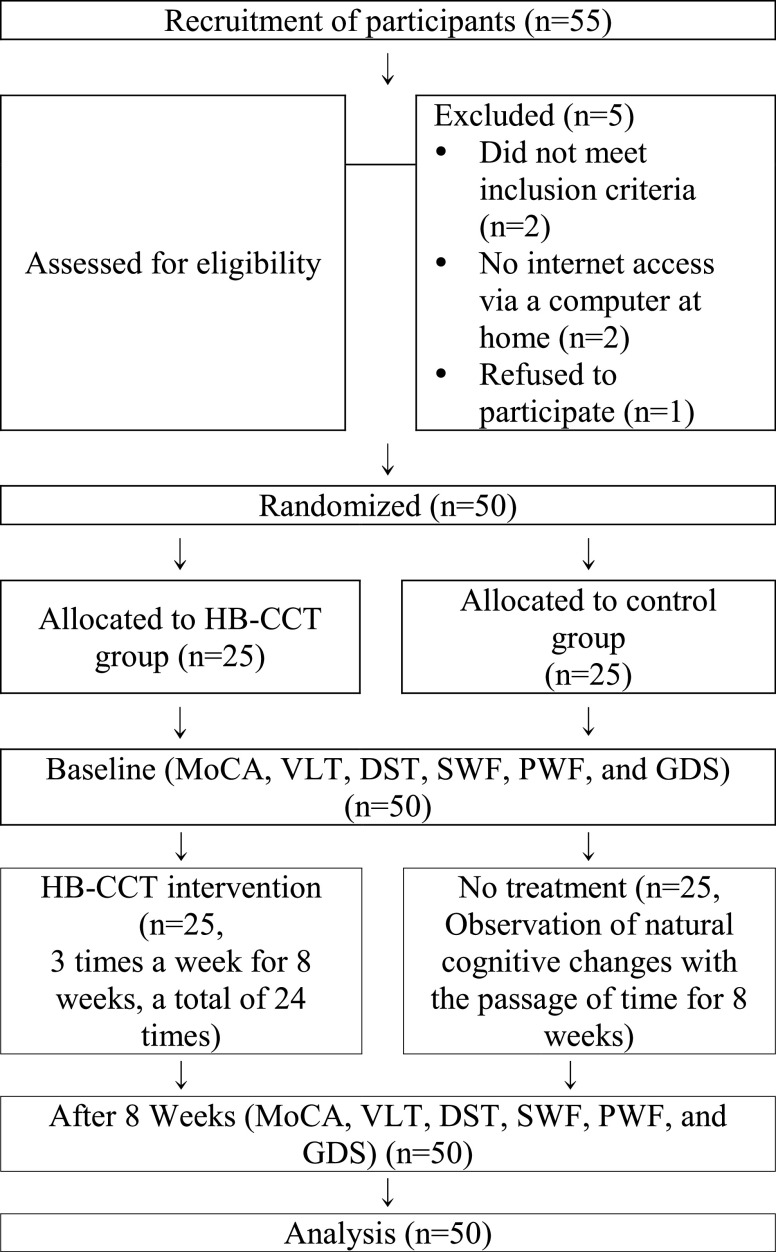
Flowchart of the study progress.

The data were transmitted immediately, and the administrator monitored the participants daily. Administrators phoned the participants each week to ensure that the interventions were without issues and to document adverse events or concomitant drug changes. The control group did not receive an intervention, and the administrator called weekly for eight weeks in order to monitor for any cognitive, environmental, or psychological changes. Treatments and medications were not restricted, and the regimen and dose that had been prescribed from the first screening visit date were maintained. The pre- and post-evaluations and distribution and collection of tablet PCs were conducted with participants visiting the hospital directly, whereas the intervention was conducted in the participants’ homes. See Figure 1 for the overview of study progress flow.

### B. Home-Based Computerized Cognitive Training

**FIGURE 2.**
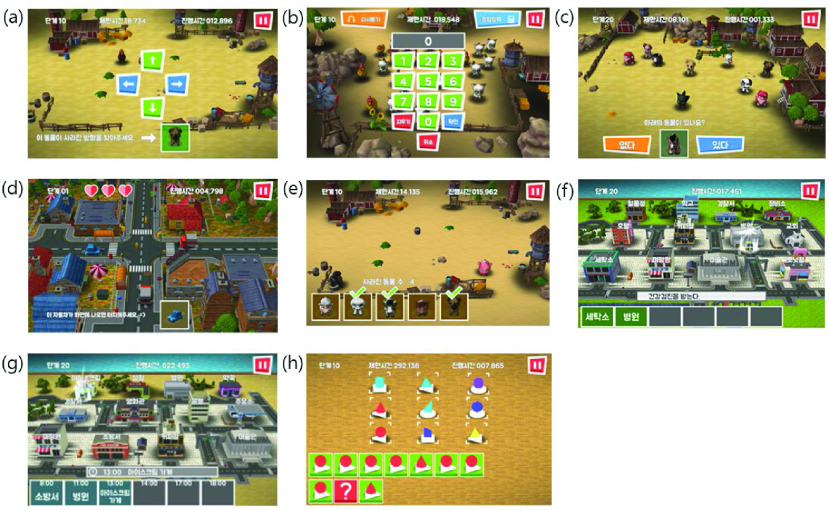
Program screen of home-based cognitive rehabilitation program (Neuro-World). (a) Sustained attention training, (b) selective attention training, (c) visual perception: exploration training, (d) visual perception: eye-hand coordination training, (e) short-term memory training, (f) working memory: work scheduling training, (g) working memory: time planning training, and (h) executive function training.

The Neuro-World (Woorisoft Inc., Daegu, Republic of Korea) HB-CCT (Version: 1.0, Server-OS: Microsoft Windows Server 2008 Enterprise SP2 (64bit), DBMS: MySQL 5.5, WAS: IIS 7, PHP: PHP 5.6, Client-OS: over Android 6.0) was used in this study. The software trains attention, visual perception, memory, and executive functions which are sub-items of cognition. The program composition and goals for each sub-category are as follows Figure 2.

Attention training was divided into two areas: sustained and selective attention training [33], [34]. In sustained attention training in Figure 2 (a), individuals were required to remember the direction in which an animal in a box had disappeared. In selective attention training in Figure 2 (b), individuals were given auditory hints and were required to correctly count the corresponding animals among various animals.

Visual perception training comprised two areas: exploration and hand-eye coordination training [35]. In exploration training in Figure 2 (c), individuals were required to choose an animal, from a multitude of animals, that was identical to an animal in a box. In eye-hand coordination training in Figure 2 (d), participants were required to select a car, among cars moving in different speeds and directions, with the same shape and color as presented in a box.

Memory training consisted of short-term memory and working memory training [36]. In short-term memory training in Figure 2 (e), participants were required to remember which of the various animals had disappeared [37]. Working memory training consisted of work scheduling and time planning. In work scheduling in Figure 2 (f), individuals needed to remember the schedule and choose pictured that described the scheduled task. In time planning in Figure 2 (g), remembering scheduled activities at specific times and choosing the picture describing that respective task were required. In executive function training in Figure 2 (h), pictures of different shapes were presented, and individuals were required to guess the shape that was likely to follow the last shown picture [38].

Each program ran for 3 min, the next training automatically commenced. Therefore, approximately 24 min was required to complete all 8 trainings. The program was designed to automatically end after completion of all 8 trainings. The hardware for performing the HB-CCT program was a tablet PC [(Samsung Co. Ltd., Seoul, Republic of Korea, Galaxy Tab S [(SM-T800]), CPU Octa-core [(Chipset: Exynos 5420 Octa]), RAM: 3 GB)]. Tablet PCs were distributed to the study participants free of charge and collected after the intervention was complete. Additionally, prior to the intervention, instructions on the use of tablet PCs and wireless fidelity (Wi-Fi) settings were provided. The initial login was performed by the administrator using the participants’ user numbers during the initial education; thereafter, the app was set up to automatically log in when clicked. Instructions and education were conducted when participants visited the hospital to receive the baseline test and tablet PC. After completing the HB-CCT intervention at home, participants visited the hospital to return the tablet PC, and post-assessment was conducted.

For HB-CCT, at-home Wi-Fi was necessary. If participants did not have Wi-Fi at home, a pocket Wi-Fi (i.e., a small portable Internet router) was provided. When a call was received from a participant who did not know how to connect the Wi-Fi to the tablet PC, the participant was provided instructions over the phone. If needed, the manager also visited the participant’s home to set up the initial Wi-Fi, after which it would automatically connect. When participants performed HB-CCT, data were sent to the manager in real time. Issues occurring during interventions were resolved through phone calls, video calls, remote access, or visits.

### C. Evaluation

The HB-CCT group underwent baseline evaluations prior to the intervention and post evaluations after the 8-week intervention. The control group underwent baseline evaluations and post-evaluations eight weeks later. The primary outcome was MoCA as an indicator of overall cognitive function [24]. The MoCA is a valid, reliable, and widely used instrument to evaluate cognitive function [39]. Additionally, secondary outcomes were verbal learning tests (VLT) [40] and digit span tests (DST) [41], [42], to assess memory and attention; semantic word fluency test (SWF) and phonemic word fluency test (PWF), to test language ability and executive function; and the Geriatric Depression Scale (GDS) [43], to assess levels of depression. In the GDS, unlike other evaluations, a lower score indicated a better result. Evaluators were blinded to maintain objectivity. The pre- and post-assessments were performed separately by different occupational therapists.

We evaluated the safety of HB-CCT by assessing whether there were any side effects such as headaches or finger pain when using it. Adverse event assessments were conducted to check for any physical issues or other discomfort related to the use of the device through weekly phone calls with participants.

### D. Usability and Adherence

To evaluate the usability and adherence of HB-CCT, we conducted observations and collected user feedback. When users encountered difficulties or issues while using HB-CCT, we identified improvement points through phone or video calls based on their feedback. Additionally, administrators monitored the usage of HB-CCT to ensure smooth usage and identify any issues. We also confirmed the participation frequency, the average training stage, and average problem-solving speed of the HB-CCT groups in each session.

### E. Statistical Analysis

Statistical analysis was performed using SPSS Statistics version 18 (SPSS Inc., Chicago, IL) and R statistical programming software package. As data from the HB-CCT and control groups followed a normal distribution with homogeneous variance, they were analyzed using parametric analysis. Paired $t$-tests were utilized to compare cognitive and depression scores at baseline and after eight weeks (with or without intervention) within each group. ANCOVA and linear mixed-effect models were used to control for baseline differences in age values and covariates, and to compare the post-evaluation scores of the HB-CCT and control groups. Significance levels were set at ${p} < 0.05$.

## III. Results

Fifty participants were included in this study, all of whom completed the study without dropout. No side effects were observed during the study period.

Table 1 provides an overview of the participants’ general characteristics. No significant differences were noted regarding ages in the HB-CCT (67.08 ± 7.92 years) and control groups (65.64 ± 8.543 years) ( $p$ = 0.619). Similarly, no significant differences were noted in the level of education ( $p$ = 0.311), between the HB-CCT (11.56 ± 1.758) and control (11.64 ± 2.289) groups.

**TABLE 1:** Participants’ General Characteristics (N = 50)

		HB-CCT group	Control group	$p$
Sex (n)	Male	8	8	
	Female	17	17	
Age (years)		67.08±7.93	65.64±8.54	0.619
Education (years)		11.56±1.76	11.64±2.29	0.311
MoCA		21.12±1.27	20.84±1.46	0.344
SVLT		15.32±4.46	15.04±4.96	0.455
DST		10.48±2.58	10.12±2.65	0.863
SWF		19.36±4.16	19.72±3.32	0.209
PWF		14.68±5.10	15.24±4.82	0.657
GDS		8.92±4.96	9.04±4.47	0.908

Data are expressed as means ± standard deviation. Abbreviations: MoCA (Montreal Cognitive Assessment), VLT (Verbal Learning Test), DST(Digit Span Test), SWF (Semantic Word Fluency Test), PWF (Phonemic Word Fluency Test), GDS (Geriatric Depression Scale), $p < 0.05$*

Table 2 shows the result of the ANCOVA analysis comparing the baseline-controlled post-evaluation results between the HB-CCT and control groups. After the 8-week intervention, the HB-CCT and control groups showed significant differences in the primary endpoint, MoCA ( ${p} < 0.01$), as well as all secondary endpoints ( ${p} < 0.05$). Among the secondary endpoints, the largest difference was seen in SWTs (F = 44.802, ${p}$ = 0.000), and the smallest difference was seen in the GDS (F = 4.952, ${p}$ = 0.031). Additional analysis using linear mixed effect models in Appendix A showed a significant difference between the two groups ( ${p} < 0.01$).

**TABLE 2:** Comparison of Baseline-Controlled Post-Evaluation Scores Between the HB-CCT and Control Groups

	SS	DF	MS	F	$p$
MoCA (R Squared = 0.479, Adjusted R Squared = 0.457)					
corrected model	$157.906^{a}$	2	78.953	21.641	0.000
Intercept	30.602	1	30.602	8.388	0.006
MoCA	20.126	1	20.126	5.516	0.023
Group	125.64	1	125.64	34.44	$0.000^{\ast} $
Error	171.474	47	3.648		
Total	24927.00	50			
corrected Total	329.380	49			
VLT (R Squared = 0.925, Adjusted R Squared = 0.922)					
corrected model	$1004.800^{a}$	2	502.400	291.086	0.000
Intercept	10.586	1	10.586	6.133	0.017
VLT	969.520	1	969.520	561.731	0.000
Group	24.944	1	24.944	14.452	$0.000^{\ast} $
Error	81.120	47	1.726		
Total	13950.00	50			
corrected Total	1085.920	49			
DST(R Squared = 0.636, Adjusted R Squared = 0.620)					
corrected model	$362.917^{a}$	2	181.458	41.041	0.000
Intercept	20.421	1	20.421	4.619	0.037
VLT	280.997	1	280.997	63.555	0.000
Group	65.944	1	65.944	14.915	$0.000^{\ast} $
Error	207.803	47	4.421		
Total	7964.00	50			
corrected Total	570.720	49			
SWF (R Squared = 0.786, Adjusted R Squared = 0.776)					
corrected model	$601.641^{a}$	2	300.820	86.064	0.000
Intercept	43.368	1	43.368	12.408	0.001
SWF	467.161	1	467.161	133.654	0.000
Group	156.597	1	156.597	44.802	$0.000^{\ast} $
Error	164.279	47	3.495		
Total	22900.00	50			
corrected Total	765.920	49			
PWF (R Squared = 0.880, Adjusted R Squared = 0.875)					
corrected model	$882.084^{a}$	2	441.042	172.747	0.000
Intercept	81.472	1	81.472	31.911	0.000
PWF	832.084	1	832.084	325.910	0.000
Group	74.009	1	74.009	28.988	$0.000^{\ast} $
Error	119.996	47	2.553		
Total	14980.00	50			
corrected Total	1002.080	49			
GDS (R Squared = 0.871, Adjusted R Squared = 0.865)					
corrected model	$789.294^{a}$	2	394.647	158.498	0.000
Intercept	8.975	1	8.975	3.604	0.064
GDS	777.774	1	777.774	312.369	0.000
Group	12.331	1	12.331	4.952	$0.031^{\ast} $
Error	117.026	47	2.490		
Total	4468.00	50			
corrected Total	906.320	49			

Abbreviations: SS (Type III Sum of Squares), DF (Degree of Freedom), MS(Mean Square), MoCA (Montreal Cognitive Assessment), VLT (Verbal Learning Test), DST(Digit Span Test), SWF (Semantic Word Fluency Test), PWF (Phonemic Word Fluency Test), GDS (Geriatric Depression Scale), $p < 0.05$*

Comparisons of the changes in the evaluation scores over time in each group is shown in Table 3. The HB-CCT group showed distinct variation across all evaluations after the intervention, including in MoCA scores (t = 4.22, ${p}$ = 0.000), our primary evaluation tool for efficacy ( $p < 0.01$). DSTs showed the largest change in value (t = 18.44, ${p}$ = 0.000), while SWFs showed the smallest change in value (t = 3.23, ${p}$ = 0.004). In the control group, scores neither decreased nor increased significantly ( $p < 0.05$).

**TABLE 3:** Comparison of Evaluation Scores at Baseline and Post (After Eight Weeks) in Each Group

	Baseline	Week-8	t	$p$
HB-CCT group				
MoCA*	21.12 ± 1.27	23.84 ± 2.21	4.22	0.00*
VLT*	15.32 ± 4.46	16.88 ± 4.00	6.19	0.00*
DST*	10.40 ± 2.57	13.44 ± 2.68	18.44	0.00*
SWF*	19.40 ± 4.22	22.68 ± 3.82	3.23	0.00
PWF*	14.72 ± 5.10	17.72 ± 4.64	9.14	0.00*
GDS*	9.08 ± 5.20	7.96 ± 4.55	3.57	0.00*
Control group				
MoCA	20.84 ± 1.46	20.52 ± 1.76	0.60	0.55
VLT	15.04 ± 4.96	15.20 ± 5.27	0.58	0.41
DST	10.12 ± 2.65	10.88 ± 3.63	1.37	0.56
SWF	19.72 ± 3.32	19.40 ± 3.43	1.22	0.18
PWF	15.24 ± 4.82	15.72 ± 4.26	1.20	0.24
GDS	9.04 ± 4.47	8.92 ± 4.07	0.82	0.24

Data are expressed as means ± standard deviation. Abbreviations: MoCA (Montreal Cognitive Assessment), VLT (Verbal Learning Test), DST(Digit Span Test), SWF (Semantic Word Fluency Test), PWF (Phonemic Word Fluency Test), GDS (Geriatric Depression Scale), $p < 0.05$*

The following are the results regarding usability and adherence. The training stage and solving speed of one training program showed significant changes during the session, as analyzed using estimated fixed effects in Appendix B ( ${p} < 0.05$). The solving speed of the participants showed a continuous significant increase up to the 8th session, followed by another significant increase at the 13th session (Appendix C). The overall significant increase in participants’ training stage was observed only up to the 6th session ( ${p} < 0.05$), and no significant increase in stage was noted after the 7th session (Appendix D). Among the 25 participants who used HB-CCT, 18 (72%) individuals completed all 24 interventions. Among the 7 participants who did not complete all the interventions, 3, 3, and 1 participants completed 20, 23, and 21 interventions, respectively. Of the total goal of 600 interventions for the 25 HB-CCT users, 582 (97%) interventions were completed.

## IV. Discussion

The results of this study indicated that the HB-CCT performed in community-dwelling adults with MCI improved cognitive function and reduced depression levels compared to the control group. As patients with MCI typically carry higher risks of developing dementia compared with healthy older adults, continuous efforts to prevent cognitive decline are necessary [10], [11]. This study provided early evidence regarding HB-CCT programs as an effective treatment for cognitive improvement in community-dwelling adults with MCI. The effects on cognitive function reported here were consistent with those reported in previous studies in which HB-CCT was used in chronic stroke patients and children with intellectual disabilities [44], [45]. Therefore, the efficacy and safety of HB-CCT may have clinical value.

A significant improvement in the MoCA, our primary outcome, was noted in the HB-CCT group compared with evaluations in the control group. Moreover, the effect level was similar to that shown by CCTs widely used in hospitals and dementia treatment institutions for cognitive therapy, such as CogReHab, COMCOG, Captain’s Log, Rehab.com, and Cog-med [12], [16], [19]. In one previous study, HB-CCT reportedly had no significant effects [46]. However, the study did not clarify whether participation was remotely monitored or whether appropriate feedback was given, as was done in our study. This may imply that monitoring treatment participation and providing feedback are essential in HB-CCT.

Regarding the secondary endpoints, short-term memory, language, and depression evaluations also showed significant improvements in the HB-CCT group compared with the control group. VLT and DST results in our study corresponded with the findings of a previous study by Klimova, and Maresova [34], [43], in which a computer or mobile phone–based cognitive intervention program may have helped improve short-term memory [47]. Training of visual-perceptual memory, target memory, selective attention, and sustained attention using the HB-CCT in this study appeared to provide positive effects in short-term memory improvement.

Additionally, SWF and PWF results in our study were consistent with a previous study that reported the ability of CCTs in improving language-related functions [48]. While the HB-CCT used in this study lacked a direct language training program, its memory, attention, and executive function training features activated the prefrontal lobe, which is likely to assist with improvements in language function [49], [50].

GDS evaluations in our study also showed results in line with previous studies that reported CCT decreasing depression levels in patients [51], [52]. Improvements in cognitive function and reduction in depression are correlated [12], [45]. Therefore, in this study, cognitive improvement may have reduced depression levels in the HB-CCT group. In addition, the program being implemented in the form of a game to allow users to have fun as well as the administration of regular feedback may have helped improve levels of depression.

In this study, HB-CCT had a positive effect on cognitive improvement and depression reduction in community-dwelling adults with MCI. All participants in this study completed the 8-week study with no drop-outs, which may have been due to the HB-CCT eliciting user interest in improving overall cognition, particularly regarding memory, attention, visual perception, and executive function. The results suggested that a tablet or mobile phone may be easily used to perform large-scale, at-home or community-based cognitive rehabilitation programs. Our results also provide evidence for the advantages in extending the temporal and spatial dimensions of cognitive rehabilitation therapies.

The HB-CCT used in this study exhibited greater portability and accessibility compared with conventional CCT programs, such as the CogReHab, COMCOG, Captain’s Log, Rehab.com, and Cogmed. Additionally, a manager was able to remotely monitor treatment participation. Based on treatment outcomes, enhanced feedback with the support of big data may also be provided, suggesting that extending the scope of CCT to homes and communities may be useful.

In this study, we found that providing accurate and specific education and solutions through immediate feedback for problems that may arise is essential in HB-CCT, as connecting to Wi-Fi or the internet, or using a new device, may not be easy for many older adults. Among the 25 participants, only 6 were able to directly connect to Wi-Fi and log in to the tablet PC; 19 participants did not know how to connect to Wi-Fi or experienced difficulties with the initial login. The administrator visited their homes on the first day of intervention to resolve these issues. However, none of the participants complained about Wi-Fi or login issues until the completion of the intervention, suggesting that even MCI adults could engage in activities using tablet PC at home with only an initial setup and minimal assistance.

The results revealed significant changes in the training stage and solving speed during the sessions. This suggests that participants’ ability to solve tasks more quickly and their overall problem-solving skills gradually increased as they progressed through the training program. However, no significant increase in the training stage was observed after the 7th session. To promote continuous growth beyond the initial sessions in the training stage, we suggest the necessity of continuously monitoring and providing feedback, as well as analyzing factors such as motivation and fatigue that can impact learning outcomes.

Among the 25 participants who used HB-CCT, 18 individuals completed all the sessions, and the training sessions achieved an overall adherence rate of 97%, indicating a strong level of commitment. This indicates the potential for sustained participation in home-based cognitive training programs. This suggests that even adults with mild cognitive impairment could engage in activities using a tablet PC at home with only an initial setup and minimal assistance. However, one limitation of this study was that the maximum training stage of the program was set to 20 and could not be increased further, which may have affected the observed lack of a significant increase in the average training stage after seven sessions. This possibly had a negative impact on the interest of the 7 participants who did not complete all the interventions in the program. The high overall completion rate of interventions suggests good engagement and commitment to the program. It is important to address the reasons for non-completion of the intervention by the participants and identify strategies to enhance adherence to future interventions.

This study holds the following clinical possibilities. First, HB-CCT can be used to expand rehabilitation therapy from hospital-based settings to community-based settings. In modern healthcare, the importance of patient-centered care and improving the quality of life has grown, leading to an increasing need for options that allow seniors and patients to establish treatment plans and receive personalized care in a home environment [53]. Therefore, further research is needed to confirm whether cognitive improvement in users continues even beyond the 8-week intervention period and whether the results are sustainable.

Second, as part of the workflow in neurology or neuropsychology clinics, HB-CCT itself can be used as a screening assessment tool or a baseline measurement. HB-CCT yields scores and performance metrics at each session, is accessible at home, and allows for continuous follow-up on its effectiveness. To achieve this, research is required to investigate whether HB-CCT’s composite scores are reliable and valid when compared to other cognitive assessments. Additionally, data collection, standardization validation, and normalization processes will be necessary, especially with a large sample.

Third, game-based HB-CCT designed based on cognitive intervention theories can be useful for enhancing user engagement and motivation. To achieve this, research is required to assess the beneficial features that HB-CCT offers to users. Furthermore, detailed evaluations and analyses are needed to determine which game formats within HB-CCT are the most effective and which types of games enhance cognitive abilities most effectively.

Fourth, because this HB-CCT uses an intuitive and user-friendly user interface, it can serve as an introductory program for older adults or patients with MCI before moving on to more complex devices or computer programs for evaluation or treatment. Therefore, research is needed to optimize the user-friendliness and accessibility of HB-CCT through various user experiences. Older adults and patients with MCI may face technological difficulties, so an analysis and research into user-friendly user interface, initial setup, monitoring support, regular visits, and user education are essential.

There were also limitations in this study. First, no intervention was given to the control group, which may have affected the motivation of individuals. Second, there may have been a learning effect that influenced the improvements, as the cognitive evaluations were repeated during the 8-week period. Third, the small sample size prevented the generalization of our results. The lack of evaluation of executive function tests, independent functional measurements, and imaging assessments could be another potential limitation of this study.

To explore these potential possibilities and address the limitations, it is crucial to conduct a randomized controlled study with a larger sample size, lasting for 8 weeks or longer for an extended intervention. It is also necessary to compare the HB-CCT and conventional CCT programs, including evaluations that were lacking in this study, to ensure the validity of our findings.

## V. Conclusion

In this study, we introduced a home-based cognitive treatment method, namely the Neuro-World, for community-dwelling adults with MCI. We have empirically demonstrated the efficacy of HB-CCT through various evaluations, including language, memory, executive function, and depression. This study proved that HB-CCT is a useful cognitive training tool for community-dwelling adults with MCI and a helpful remote feedback tool for therapists and professionals in hospitals. We also suggested ways of reducing HB-CCT failure in older adults. As the next step of this study, a larger randomized controlled trial comparing HB-CCT with other CCT programs would be necessary.

## Appendix A

See Table 4.

**TABLE 4:** Linear Mixed-Effect Model Results for Each Groups According Times

Fixed effect	Estimate	SE	DF	t	$p$
MoCA					
Intercept	20.520	0.343	87.963	59.905	0.000
Group	3.320	0.484	87.963	6.853	0.000
Time	0.320	0.405	48.000	0.791	0.433
Group*Time	−3.040	0.572	48.000	−5.312	0.000
SVLT					
Intercept	15.200	0.940	49.929	16.176	0.000
Group	1.680	1.329	49.929	1.264	0.212
Time	−0.160	0.264	48.000	−0.606	0.547
Group*Time	−1.400	0.373	48.000	−3.752	0.000
DST					
Intercept	10.880	0.583	61.850	18.665	0.000
Group	2.560	0.824	61.850	3.106	0.003
Time	−0.760	0.418	48.000	−1.819	0.075
Group*Time	−2.280	0.591	48.000	−3.858	0.000
SWF					
Intercept	19.400	0.743	55.228	26.115	0.000
Group	3.280	1.051	55.228	3.122	0.003
Time	0.320	0.394	48.000	0.812	0.421
Group*Time	−3.600	0.557	48.000	−6.457	0.000
PWF					
Intercept	15.720	0.942	51.502	16.680	0.000
Group	2.000	1.333	51.502	1.501	0.140
Time	−0.480	0.354	48.000	−1.357	0.181
Group*Time	−2.520	0.500	48.000	−5.036	0.000
GDS					
Intercept	8.920	0.918	51.675	9.719	0.000
Group	−0.960	1.298	51.675	−0.740	0.463
Time	0.120	0.353	48.000	0.340	0.735
Group*Time	1.000	0.499	48.000	2.005	0.051

Abbreviations: SE(Standard Error), DF(Degrees of Freedom), ${p}< 0.05$*

## Appendix B

See Table 5.

**TABLE 5:** Linear Mixed-Effect Model Results for Speed per Item and Training Stage of HB-CCT Group

Fixed effect	numDF	denDF	F	$p$
Average speed per item				
Intercept	1	558	3046.609	0.000
Time	23	558	4.072	0.000
Average training stages				
Intercept	1	558	4491.455	0.000
Time	23	558	7.195	0.000

Abbreviations: num(numerator), den(denominator), DF(Degrees of Freedom), ${p}< 0.0$*

## Appendix C

See Table 6.

**TABLE 6:** Estimates of Fixed Effects b of Linear Mixed-Effect Model Results for Speed per Item of HB-CCT Group

Parameter	Estimate	SE	DF	t	$p$
Intercept	5.865	0.793	558.000	7.395	0.000
[TIME=1]	6.349	1.040	558.000	6.104	0.000
[TIME=2]	4.398	1.040	558.000	4.229	0.000
[TIME=3]	2.078	1.040	558.000	1.997	0.046
[TIME=4]	3.242	1.040	558.000	3.117	0.002
[TIME=5]	2.153	1.040	558.000	2.070	0.039
[TIME=6]	2.551	1.040	558.000	2.453	0.014
[TIME=7]	2.665	1.040	558.000	2.562	0.011
[TIME=8]	2.101	1.040	558.000	2.020	0.044
[TIME=9]	1.783	1.040	558.000	1.714	0.087
[TIME=10]	1.998	1.040	558.000	1.921	0.055
[TIME=11]	1.802	1.040	558.000	1.733	0.084
[TIME=12]	2.003	1.040	558.000	1.925	0.055
[TIME=13]	2.260	1.040	558.000	2.173	0.030
[TIME=14]	1.372	1.040	558.000	1.319	0.188
[TIME=15]	0.832	1.040	558.000	0.800	0.424
[TIME=16]	1.660	1.040	558.000	1.596	0.111
[TIME=17]	1.199	1.040	558.000	1.153	0.249
[TIME=18]	1.307	1.040	558.000	1.256	0.210
[TIME=19]	0.909	1.040	558.000	0.874	0.383
[TIME=20]	0.941	1.040	558.000	0.904	0.366
[TIME=21]	0.724	1.069	558.000	0.677	0.499
[TIME=22]	0.008	1.081	558.000	0.008	0.994
[TIME=23]	0.275	1.081	558.000	0.254	0.799
[TIME=24]	0.000	0.000	.	.	.

Abbreviations: SE (Standard Error), DF (Degrees of Freedom), $p < 0.05$*

## Appendix D

See Table 7.

**TABLE 7:** Estimates of Fixed Effects b of Linear Mixed-Effect Model Results for Training Stage of HB-CCT Group

Parameter	Estimate	SE	DF	t	$p$
Intercept	12.606	0.927	558.000	13.599	0.000
[TIME=1]	−8.526	1.216	558.000	−7.013	0.000
[TIME=2]	−5.768	1.216	558.000	−4.745	0.000
[TIME=3]	−4.598	1.216	558.000	−3.782	0.000
[TIME=4]	−3.477	1.216	558.000	−2.860	0.004
[TIME=5]	−2.677	1.216	558.000	−2.202	0.028
[TIME=6]	−2.468	1.216	558.000	−2.030	0.043
[TIME=7]	−2.232	1.216	558.000	−1.836	0.067
[TIME=8]	−1.947	1.216	558.000	−1.602	0.110
[TIME=9]	−1.466	1.216	558.000	−1.206	0.228
[TIME=10]	−1.164	1.216	558.000	−0.957	0.339
[TIME=11]	−1.106	1.216	558.000	−0.910	0.363
[TIME=12]	−1.156	1.216	558.000	−0.951	0.342
[TIME=13]	−0.836	1.216	558.000	−0.688	0.492
[TIME=14]	−0.688	1.216	558.000	−0.566	0.572
[TIME=15]	−0.585	1.216	558.000	−0.481	0.631
[TIME=16]	−0.548	1.216	558.000	−0.451	0.652
[TIME=17]	−0.328	1.216	558.000	−0.270	0.787
[TIME=18]	−0.243	1.216	558.000	−0.200	0.841
[TIME=19]	0.102	1.216	558.000	0.084	0.933
[TIME=20]	0.176	1.216	558.000	0.145	0.885
[TIME=21]	0.097	1.250	558.000	0.078	0.938
[TIME=22]	−0.090	1.263	558.000	−0.072	0.943
[TIME=23]	0.061	1.263	558.000	0.048	0.962
[TIME=24]	0.000	0.000	.	.	.

Abbreviations: SE (Standard Error), DF (Degrees of Freedom), $p < 0.05$*

**Conflicts of interest**—There are no conflicts of interest to declare.
